# From uncertainty to gradually managing and awaiting recovery of a periodic condition- a qualitative study of parents´ experiences of PFAPA syndrome

**DOI:** 10.1186/s12887-019-1458-y

**Published:** 2019-04-08

**Authors:** C. Sparud-Lundin, S. Berg, A. Fasth, A. Karlsson, P. Wekell

**Affiliations:** 1 Institute of Health and Care Sciences, Sahlgrenska Academy, University of Gothenburg, Box 457, SE-, 405 30 Gothenburg, Sweden; 2 Department of Pediatrics, Institute of Clinical Sciences, Sahlgrenska Academy, University of Gothenburg, Gothenburg, Sweden; 3 Department of Rheumatology and Inflammation Research, Institute of Medicine Sahlgrenska Academy, University of Gothenburg, Gothenburg, Sweden; 4 Department of Pediatrics, NU-Hospital Group, Uddevalla, Sweden; 5 Department of Pediatrics, Institute of Clinical Sciences, University of Gothenburg, Gothenburg, Sweden

**Keywords:** Periodic fever, PFAPA, Parents experiences, Grounded theory

## Abstract

**Background:**

The prevalence of periodic fever, aphthous stomatitis, pharyngitis and cervical adenitis (PFAPA) syndrome is unknown. Although an uncommon condition, it is considered to be the most common autoinflammatory disease among children in many parts of the world. The knowledge of the consequences of the recurrent fever episodes for the child and its family are limited. This study explores the experiences of parents regarding the impact of the disease on the child’s general well-being, the family’s situation and how the family handles the associated challenges.

**Methods:**

A qualitative approach was used, applying a modified version of Grounded theory for design, data collection and analysis. Data was collected from two different sources: communication between parents of children with PFAPA in a closed Facebook group and face-to face interviews with one of the parents of children diagnosed with PFAPA (6 mothers and 2 fathers).

**Results:**

Parents described a lengthy process of how everyday life becomes affected by their child’s recurrent fever episodes. This process is depicted in the following Grounded Theory core category: From uncertainty to gradually managing and awaiting recovery. The categories Uncertainty, Assurance, Gradually managing and Recovery describe the experienced illness trajectory. The illness representation illustrates the experiences/impacts of the periodic condition in the subcategories: Harmlessness-Severity, Disclosure of diagnosis, Impact on daily life and Regularity-Unpredictability. The children’s well-being was highly affected by the symptoms during episodes. Parents experienced increased stress with constant fatigue, social constraints of family life and restricted career opportunities. Nevertheless, hope of recovery was constantly present.

**Conclusions:**

PFAPA is associated with a considerable burden on the child and the parents in daily life. Obtaining a diagnosis enables parents to move from a state of uncertainty towards a sense of coherence while awaiting recovery. Because of limited general knowledge of the condition and its impact on daily life, health care professionals need to become aware of the parents’ efforts to mitigate the consequences of the recurrent episodes for the child and for the family as a whole.

## Introduction

Children who suffer from periodic fever, aphthous stomatitis, pharyngitis and cervical adenitis (PFAPA) syndrome have recurrent attacks of fever associated with symptoms from the throat for several years [1]. Typically, the attacks are regular, in intervals of 3–8 weeks and durations of 3–6 days. Between episodes, children with PFAPA are by definition completely asymptomatic, with normal growth and development [2]. Despite a good long-term prognosis for children with PFAPA syndrome, our clinical experience indicates that the disease substantially influences the child’s general well-being and the family situation as a whole.

PFAPA episodes are symptomatically treated with NSAIDs and paracetamol. In some parts of the world, each episode is treated with corticosteroids, as they efficiently abort an attack. However, treatment with corticosteroids may shorten the intervals between episodes [3]. A positive response to corticosteroids is sometimes used as additional support for the PFAPA diagnosis [3]. In Sweden, corticosteroids are primarily used to postpone a febrile episode that is occurring at a particularly unsuitable time for the child or the family [4]. Tonsillectomy has turned out to be an attractive treatment alternative in many cases [5–8]. The prevalence of periodic fever, aphthous stomatitis, pharyngitis and cervical adenitis (PFAPA) syndrome is unknown. Although it is a rare condition, it is considered to be the most common autoinflammatory disease among children in many parts of the world. The only data on epidemiology is from Norway, with an approximated yearly incidence of 2.3 per 10,000 children up to 5 years of age [9]. Although many parents recognize that the disease substantially influences the situation of the child and the family as a whole, the impact of the disease in this respect has not been studied in a systematic way. This study aims to explore the experiences of parents of children with PFAPA regarding impact on the child’s general well-being, family life situation and how the family manages the challenges that accompany the condition. Based on such an in-depth understanding, the study can provide new insights that can be used to meet the healthcare needs of affected children and their families, and serve as an impetus for further studies.

## Methods

A modified version of Grounded Theory (GT) was used [10]. This methodology is useful for exploring how people learn to manage new or chronic health conditions [11]. In this study, we used 1) internet communication between parents of children with PFAPA and 2) face-to face interviews with parents of children with PFAPA in order to further explore main issues.

### Sample and data collection by internet mining

A Facebook group directed to people with common interest in PFAPA was identified as a source of information. The PFAPA Facebook group is a closed group, initiated in September 2011 and currently involving around 3500 members, mainly North American inhabitants. The web-community contains rich data with an interactive forum where parents discuss their experiences of having a child with PFAPA. All communication posted over a period of three different months (February, April, and October) in 2013–2014 was collected in order to capture potential seasonal variation in experiences. The posts were copied and pasted into a Word-document for further text analysis.

### Sample and data collection by interviews

Face-to face interviews were conducted with 8 Swedish parents (6 mothers and 2 fathers) of children with typical features of PFAPA syndrome, defined as 1) fulfilment of the standard clinical criteria for PFAPA syndrome [2], 2) lacking additional features suggestive of hereditary periodic fever syndromes or, an unclassified periodic fever syndrome [12]. The parents were consecutively recruited from two paediatric outpatient clinics in western Sweden (see Table 1 for demographic information). The physician consecutively approached the parents during their child’s consultation, gave information about the study and obtained consent to participate in an interview. All respondents consented to participation. The interviews were conducted by a researcher not involved in paediatric health care, in a place chosen by the informant. Each interview followed an interview guide with open-ended questions and lasted between 30 and 70 min. The questions focused on how the parents perceived the impact of the disease on the general well-being of their child and on the situation of the family as a whole, as well as how the family manages the challenges that accompany the condition. Follow-up questions were asked in order to obtain rich descriptions. The interviews were digitally recorded for subsequent verbatim transcription.

**Table 1:** Demographic data on parents (*n* = 8) and their children with PFAPA syndrome

Age median *(*range*)*	
*Parents’ age at interview (years)*	35.5 (29–43)
*The child’s age at interview (years)*	4.0 (2–7)
Tonsillectomy (n)	2
Education (*n*)	
*Secondary school*	3
*University level*	5
Marital status (*n*)	
*Living with the other parent*	7

### Analysis

**Fig. 1:**
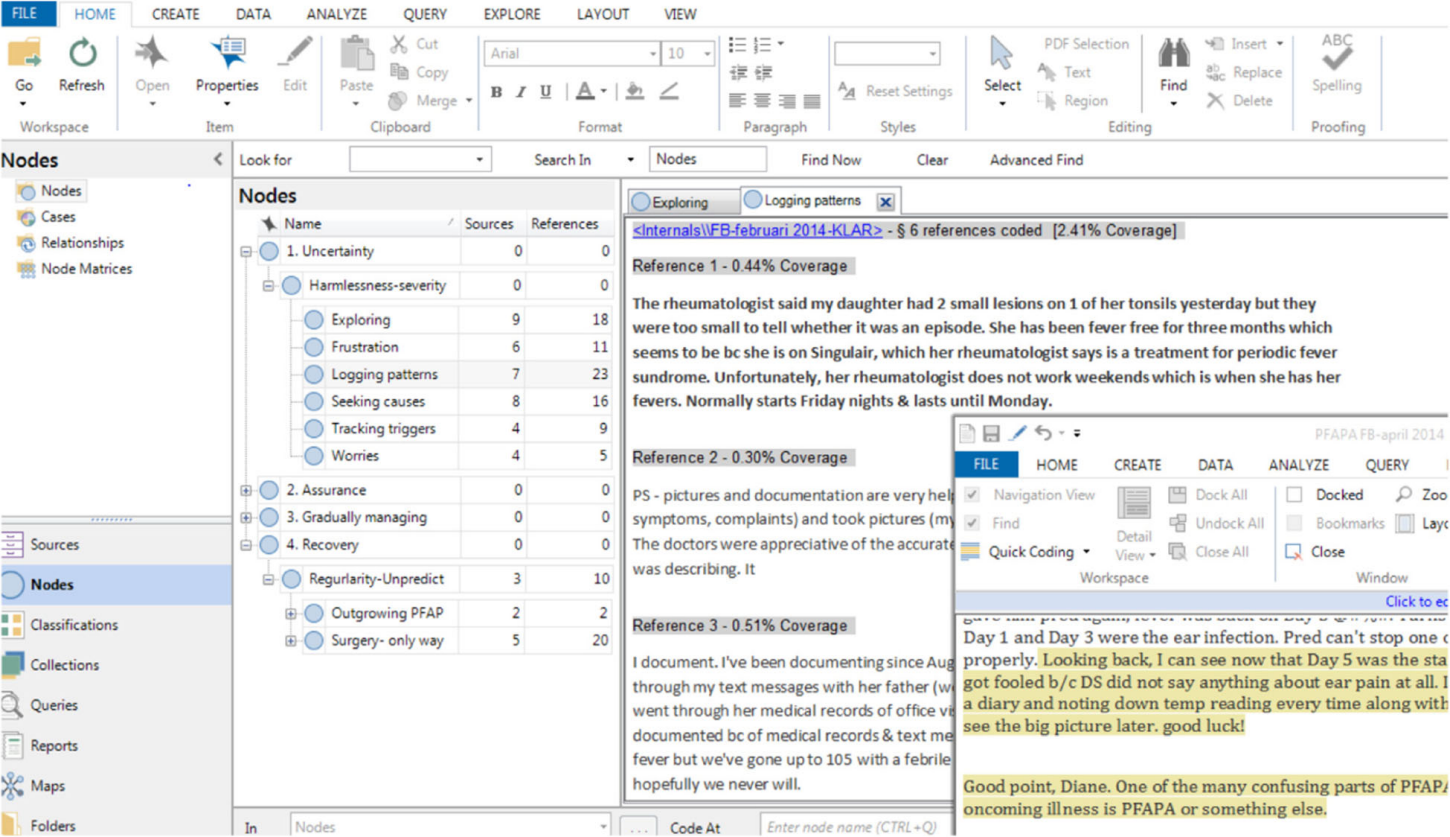
Screen-shot showing an example of the qualitative computer assisted data analysis (Nvivo 11 Pro)

The analysis was inspired by the Qualitative Guide of Analysis (QUAGOL) [10]. The preparation coding process included reading the PFAPA Facebook group’s posts to get a holistic understanding of their experiences. Interviews were processed similarly, with each interview summarized in a narrative memo to capture the core content and meaning within its context. A preliminary conceptual data scheme was developed based on concrete experiences using constant comparison technique. In subsequent steps, a list of concepts was drawn up as preliminary codes by using QSR International’s NVivo 11 Software. The text was linked to relevant codes and the concepts were analysed by identifying their characteristics, meaning and dimensions. Linking of categories, general patterns, similarities and differences was continuously sought by constant comparisons within and between codes and categories by two of the authors. Selective coding was used to saturate the categories and theoretical sampling was conducted to achieve saturation of all categories generated in the analysis (example of the analytical process in Fig. 1). High replication of data within emerging categories and verification of experiences and strategies by the participants indicated saturation. Written memos were used to capture key findings. Finally, we developed a conceptual framework to depict the essential structure of parents’ experiences and their strategies to manage their life situation.

## Results

**Fig. 2:**
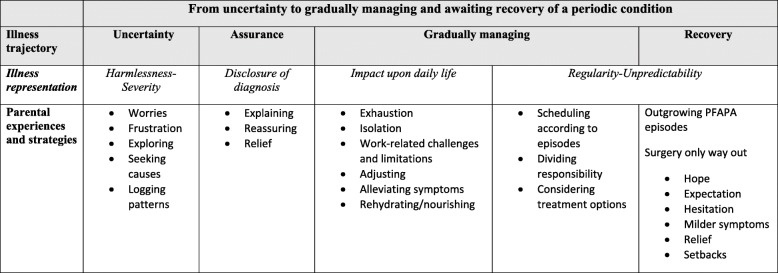
Conceptual model depicting the process of illness trajectory and representation reflecting parents’ experiences of PFAPA

A conceptual model illustrating the illness representation and trajectory of parents’ experiences of the impact of PFAPA and their strategies to manage the challenges is represented in Fig. 2. Parents described a lengthy process, depicted in the following core category: *From uncertainty to gradually managing and awaiting recovery***.** The first phase in the illness trajectory was characterized by uncertainty when parents alternately hoped for a harmless reason for their child’s condition or feared a more severe cause of the frequent fever episodes. The parents commonly handled this uncertainty by exploring and logging symptoms and patterns in order to seek causes of the fever. Some assurance was obtained with the disclosure of the diagnosis, resulting in relief and explanation, which helped the parents deal with their own frustration and that of others. The child’s health and well-being were reported to be highly affected by the recurrent episodes and absence from activities sometimes led to social isolation and academic shortcomings. For the parents, increased stress from everyday life resulted in constant fatigue, social constraints of family life and a negative impact on career opportunities. The parents gradually learned to manage the fever episodes and their consequences by dividing responsibility between them, and carefully preparing and planning for the anticipated episode. The hope of a recovery was constantly present, as the parents knew that the child could grow out of the disease, or improve after tonsillectomy.

Below, the process reflecting the parental experiences and strategies is described in detail with main categories describing the illness trajectory (in bold) and subcategories illustrating the illness representation (in italics), Quotes are cited as I for interview and number (I:1–8) and (FB) for communication in the Facebook community.

### Uncertainty

#### Harmlessness – severity

During the phase of uncertainty, parents described how their feelings and worries about the child’s condition and potential diagnosis switched between hope of harmlessness to fear of severity. Their worries also included side effects of the antipyretics and antibiotics given, as well as the discomfort and possible complications of frequent physical examinations, investigations and blood samplings. Parents described the situation as frustrating due to the lack of knowledge about PFAPA within the health care system. Sometimes health care providers dismissed the child’s frequent febrile episodes as being a normal pattern of infections for a toddler.

*“And we kinduv’ felt…how much antibiotics should we give her? How will it affect her? On top of all the Alvedon (paracetamol) and Ipren (ibuprofen) we’re were doing all the time. And during that period we were…there was a lot of worrying and wondering like ʻis it something serious?’ and you’d ask the question ʻwhat on earth can it be? Is there something wrong with her or has she got a tumour?”(I:8).*

This phase of uncertainty involved exploring and seeking causes of the fever episodes. Some parents searched for potential diagnosis on the internet, and eventually found PFAPA to be a possible one.

*“So then we started to think about it ourselves, ʻcan it be…the fever system…can you have something wrong with it? So that you get a fever although you’re not sick?’ But we didn’t have the slightest idea what diagnoses there were – and it wasn’t easy to find. Then you google a bit and find something called periodic fever and recognised yourself a little in that.” (I:1).*

*Sometimes I wonder if it is related to PFAPA. My daughter usually has fever for 4–5 days and symptoms last 7–9 days. She is only 16 months so we are still in observation stage and trying prednisone next episode. (FB).*

*I am guessing almost every time. Never quite sure whether this is PFAPA or something else just starting. My daughter fevers also come back a couple of days after Pred but it has never been anything else - no other “normal” disease. (FB).*

Parents described how they were logging patterns on their own initiative. Others were encouraged by health care workers to keep a fever log book, which was appreciated when they came to the health care visit prepared with information about symptoms and fever intervals. For some parents, the active strategies of logging seemed to help them handle the uncertainty, contributing with information that could potentially solve the “mystery” of the child’s condition.

*I have a blog that I write in a bit privately only then. And it was…after some months I started to feel…and then the fever again and then it was gone and I went back to see what I’d written. It was a bit like a diary too. Then I started to put together what I’d written in the blog and then I realised that it was still the same pattern here. So that’s when we started to wonder...(I:3).*

### Assurance

#### Disclosure of diagnosis

Receiving a diagnosis was very important for the parents. People in general are largely unaware of the condition, and the diagnosis helped the parents to regain credibility after all their absences from work. The diagnosis explained their situation and a certificate from the physician enhanced managers’ and colleagues’ understanding and patience.

*It’s frustrating to try to explain to people sometimes because they just don’t understand what it’s like and that it’s a real illness. (FB).*

*Being able to get a certificate, it was such a relief to be able to give it to the principal and even my husband’s employer and the social security office. So that…we’re not doing this because it’s fun or we’re lazy but so we could explain to other people and get them to understand at work and at the social security office and the like. (I:2).*

Having the child finally diagnosed with PFAPA was described as reassuring. The parents had feared so many other more serious or life-threatening conditions and this diagnosis gave hope for the future. With this reassuring knowledge and their extensive experience of caring for the child during the fever episodes, the parents became experts at managing the condition.

*But all of a sudden we found ourselves in a situation where we actually are the experts in a way. (I:4).*

Especially after a long history of symptoms, often fluctuating over time, one notable experience was the feeling of relief that came with an established diagnosis:

*My daughter just turned 12 and was diagnosed with this this past week. She has been experiencing symptoms since she was about 15 months old. I feel so relieved to finally have some answers but also sad that it took so long to figure out. She has had fever free periods in her life but for the most part she gets quite sick every few weeks. (FB).*

### Gradually managing

#### Impact upon daily life

The parents described how the periodic fever impacted upon the child and themselves in different ways. The child’s suffering*,* as described by the parents*,* was mainly related to PFAPA symptoms during the episodes. A general impaired condition, difficulties in feeding the child and sudden changes owing to symptoms like vomiting caused practical problems. This resulted in parents worrying about the long-term effects on their child’s physical wellbeing and weight development.

*I have to go round carrying him all the time because I…yes, among other things I want to keep an eye on him because I know that too high a temperature is no good. If he needs to go to the toilet – or if I need to – I have to take him with me, because he doesn’t have the energy and he’s really miserable and tired. (I:7).*

The parents felt they needed to be prepared to treat the child with antipyretics to avoid fever-related symptoms. Some parents reported their child suffering from impaired general health between the episodes, owing to the short time lapses for recovery. The children’s mood seemed to be more or less affected the days before a fever episode but also some days after.. Moreover, the fever could be followed by decreased appetite and activity. Parents tried to use the time between episodes to compensate for insufficient nutritional intake during episodes. Parents also perceived the condition as having a social impact on the child’s daily life, as recurrent absences from daily activities, nursery school, school and leisure activities could lead to impaired social interaction and isolation.

*Because he’s ill so much, and starting a new class, it affects him a lot socially. Because as soon as he starts to play with someone, he’s away for a week, so it feels like you kind of become a bit unreliable. It can be difficult to get in with a group of friends. (I:1).*

Moreover, parents stated that the family could become more or less isolated due to frequent cancelling of social events, but also because of the exhaustion that followed the child’s recurrent illness episodes. In the Facebook group, parents shared their experiences of exhaustion with each other:

*People, the pediatrician and specialist always try to be sweet and say he will most likely outgrow it so as to say, no worries. But how much can a young child really bear and how much can a mother/ family take? (FB).*

*Gets so exhausting, our little guy gets it every 19 days for 5–6 days so we usually only get a 14 day break. I really feel like I am drowning lately. (FB).*

Parents experienced work-related challenges in terms of dealing with frustrated colleagues and managers who had to replace them during recurrent sick-leaves. For some parents, it became more or less impossible to maintain their jobs. In the Facebook community, parents discussed how they tried to solve this unsustainable situation in daily life:

*My question for everyone is how do you manage time out of work? I’ve worked only 2 full weeks since mid-January and hearing this may be our new normal until she grows out of it. It scares me. Anyone have to quit work because of the frequency? (FB/February).*

*Fortunately, I stay home. There is no way I would be able to hold a position with him being sick so often. My husband was traveling often for work and had to take another position. (FB).*

Parents also shared stories of missed opportunities in their professional career because of the consequences of their child’s disease. For example, in order to manage the family situation, they hesitated to take on a career opportunity or considered leaving a position they had already reached.

*He (the father) had just got a new job – a new position as head of projects – before she got ill but…he felt he had to be at home a lot because I…in the beginning I was at home the most but then he had to be at home a lot too; we had to start sharing it, so he felt that he couldn’t fulfil his duties that well…that he had hit the wall.* (I:2).

As described in the quote above, parents had to adjust to solve these problems in daily life, and in so doing, they were at risk of health problems themselves. Work-related challenges also appeared to be problematic even when one of the parents was on parental leave or a ‘stay at home’ parent. Although many parents described a lack of understanding among employers and colleagues, some experienced more considerate and accepting behaviours in their workplace:

*They’ve even been able to say to me: ʻSo when is your child going to be sick again? Because we’re planning the schedule now’ (I:7).*

#### Regularity – unpredictability

Most parents described the fever episodes as appearing with a certain regularity. Although the child’s condition and its consequences were frustrating and problematic, this regularity was sometimes helpful in daily life, since activities and events could be planned according to the episodes.

*Yes, there’s between two, three weeks (between fever episodes). But for a while it was like that – because I work in a school – so it was like: ʻthis week it’s been good so Wednesday, Thursday… so sometime next week I can count on going home.’ So then it was like you could think about it beforehand (I:5).*

*It’s nice to know I’m not the only one that has a Friday- Monday child, she usually starts Friday evening which is too late to drive her to Boston. I don’t work Fridays either so it would be perfect if she started on Thursday night instead, ha-ha (FB).*

Others experienced a more unpredictable occurrence of fever episodes. The illness could change from a regular to a more unpredictable pattern over time. The practical advantages of the regular pattern became evident when this regularity was interrupted by treatment with steroids, or when the child got a viral infection between the episodes, which in both cases hampered planning.

During the interviews, the parents described how they resolved sick leave duty during episodes by either sharing the responsibility equally, for example by taking every other day, or by deciding according to assignments at work, workload, financial consequences and/or any other practical reason. Shared responsibility was less evident in the international context of Facebook interactions, where it was mostly mothers interacting and commenting that they took primary responsibility for the care of the child during episodes.

*But we try to work it out and see who has the most to do, who’s finding it the most difficult to get away. So that we still have…I’ve probably taken on more because I’ve been free one day a week, but he’s been home absolutely – yes almost half the time I’d say. (I:2).*

*The fever started in the morning, it wasn’t an evening thing – she had fever when we woke up and by then my hubby had already gone to work so I was the one who stayed at home. And it was the financial situation we thought too: he earns a lot more than me and so we benefit from him going to work. I’m used to taking care of kids…we haven’t even talked about who should stay at home, it’s usually me. (I:5).*

What was a sometimes unsustainable situation for the child and the family led to considering more active treatment options such as steroids and eventually surgery, i.e., tonsillectomy. Parents appeared to be aware that steroids could be used to abort a single episode at an unsuitable time, but many expressed doubts about the effects and consequences of this alternative. Sometimes they described how steroids could shorten the interval between episodes.

*My little boy will be 2 on Friday, and his tonsillectomy is next Thursday, I am uneasy about it but our life is not a life anymore. Steroids have also brought fevers closer, every 2 weeks or less. (FB).*

### Recovery

#### Regularity – unpredictability

In the recovery phase, the illness was still representing itself in terms of regularity and unpredictability. The parents hoped and expected that their child would outgrow the PFAPA episodes as he or she became older, as described by health care providers or other parents with such experiences. However, uncertainty as to if and when this would occur gave rise to feelings of unpredictability and frustration, and made it difficult to know what to expect of the future.

*She’s getting big now so…they also say that it seems they’ll outgrow or it’ll turn into something else but I don’t know much more about it…and then even age…we’ll have to see what becomes of it later. If there are more symptoms or something like that, we don’t know. (I:6).*

After a period of less frequent episodes, parents continued to keep track of the fever pattern to know if the end of PFAPA episodes was approaching, otherwise they felt they would have to consider tonsillectomy instead of waiting for their child to outgrow the disease.

*He has not had an episode since DECEMBER. This is monumental for us. (FB/February).*

He *seems* to be outgrowing it. Episodes are further between, and generally much less severe. Current plan is to hold off on considering T & A (tonsillectomy and adenoidectomy) *if this continues. Fingers crossed. (FB).*

For some parents, tonsillectomy was considered the only way out of this unmanageable situation. In the Facebook community, discussion largely focused on worries about the surgical procedure, for example, surgical complications, postoperative suffering for the child, other negative consequences, and if surgery had been curative or not.

*Doing it felt like the only way out for us when things were so difficult for us, that we wanted it more than anything, and then it was the age limit of three that prevented us from doing it earlier. So when he was three in March, he (the doctor) got us a referral. (I:3).*

There were many success stories among the parents, who justified their decision by comparing daily life before and after surgery, sometimes encouraging others to take the decision, as illustrated in the following communication:

*Our Tonsillectomy was a cake walk compared to how crappy the PFAPA episodes were.*

*-Agree. Tonsillectomy recovery was no fun but so much better than episodes every two weeks.*

*- I would do tonsillectomy again in half a heartbeat. It was the right decision for us*. (FB).

*A tonsillectomy gave us a 3 year remission period. Even though the fevers came back, the surgery was worth it to have 3 “normal” years! (FB).*

Although many children seemed to be more or less symptom free after surgery, others who had got rid of the fever still had regular ‘ghost’ episodes, which were mostly milder in terms of frequency and severity. Symptoms could be combinations of tiredness, abdominal pain, mouth ulcers, crankiness or minor problems with a sore throat. In those cases, the milder symptoms made life less unpleasant for the child and more manageable for the parents.

*Primarily, it (the tonsillectomy) meant he could eat and drink better during his episodes and even if he…vomited it comes up. Before it he was so swollen in his throat because it was pretty slimy then, it just got stuck in the throat. He had a lower temperature than what he used to and its’s a bit easier to manage as well. (I:3).*

However, compared to before surgery, these symptoms had less impact on general health and fewer social consequences. In the Facebook community, parents also shared stories about setbacks after the child was supposed to have outgrown their illness. Having felt such relief following the child’s recovery, the parents were clearly disappointed by the return of symptoms.

*Wondering if anyone else has had the same thing? My daughter had PFAPA when she was about 5 and it lasted for about 3 years. I thought it was over and we were past it when she started getting the symptoms again at age 13 coincidently after her menstrual cycle started. (FB).*

## Discussion

This qualitative analysis is based on the parents’ experiences of having a child with PFAPA syndrome. The findings contribute knowledge of how everyday life becomes affected during a process with different phases; from a state of uncertainty to assurance after disclosure of the diagnosis, and how the parents gradually learn to manage their daily life while waiting for either spontaneous recovery or tonsillectomy. In the illness trajectory, the parents experienced regularity-unpredictability in both the phase of gradually managing and longing /hoping for recovery. This was related to fever episodes as well as the potential out-growing/ healing after tonsillectomy. (Fig. 2).

The key to the diagnosis of PFAPA relies on the recognition of recurrent regular febrile episodes with the same clinical features, although a single episode is difficult to distinguish from an infection with certainty. Limited awareness of the condition among healthcare providers along with diagnostic challenges often result in a long period without a proper diagnosis, characterized by repeated healthcare visits accompanied by blood sampling, investigations and antibiotic treatments. In this study, interviewed parents and members in the Facebook community describe the uncertainty, the frustration and the worries before their child was diagnosed with PFAPA. These pre-diagnostic experiences are similar to that of parents whose children are subsequently diagnosed with more severe conditions, for example, malignancies or Kawasaki disease [13]. On the other hand, the diagnostic disclosure of PFAPA resulted in psychological relief and provided the parents with an explanation – an experience that is in sharp contrast to the overwhelming uncertainty that parents of children with a life-threatening condition have to live with [14].

When the child’s condition was diagnosed as PFAPA it made the situation more understandable, and allowed parents to gradually learn to manage their daily life by dividing responsibilities in a more conscious way by planning their activities with episodic regularity in mind, and carefully preparing for the anticipated episode. This may explain our clinical observation that many parents did not express a need for regular follow-up visits after the disease was diagnosed, if they could contact the physician when needed. This can also be linked to the concept of sense of coherence, where the three dimensions comprehensibility, meaningfulness, and manageability [15] are reflected in the importance of obtaining the PFAPA diagnosis, which in turn helps parents to develop strategies to manage everyday life.

In PFAPA syndrome, the fever episodes reoccur for approximately 3–5 years with variations in frequency, length and intensity. In this study, the parents made clear that they had spent years constantly waiting and hoping their child would recover, either by outgrowing the disease or improving after tonsillectomy. In the interview cohort, possible improvement after tonsillectomy was delayed due to clinical practice in Sweden, which recommends tonsillectomy after the age of three. The reasons for this are that the immunological consequences of tonsillectomy are unknown at a very early age and that adenotonsillectomy for obstructive sleep apnea syndrome has been associated with a greater risk of postoperative respiratory complication in children under the age of three, as compared to older children [16]. The results in the interview study made the clinicians in the research group reconsider their clinical practice for children below the age of three, by offering the family a trail of colchicine treatment and starting a process of reevaluating the scientific support for the recommended age limit for tonsillectomy.

Parents reported substantial impact on their work situation due to the frequent fever episodes. Shared responsibility between parents seemed to be more common in the interviews (the Swedish context) than in the Facebook community, where most participants appeared to be US citizens. This is not very surprising since Swedish governmental policies include a greater allowance for combining parenthood and employment and a more generous reimbursement for care of sick children than many other countries, which is likely to support shared responsibility for sick leave. Such differences were also found in a qualitative study with parents of children newly diagnosed with type 1 diabetes [17]. However, in the present study it was more evident that the career development of the parents was adversely affected by the recurring absence periods. This indicates a significant impact on the parents’ overall life situation.

In the classical definition of PFAPA syndrome, one of the criteria is normal growth and in clinical practice, children rarely (most often with very frequent episodes) that lose weight. This study shows that normal growth seems to be achieved by a combination of significant parental effort and the child’s increased appetite between episodes.

### Methodological considerations

We used two data collection methods in this study, which needs to be acknowledged. The objective was to capture both the broad picture of parents’ experiences in the Facebook community and a deeper understanding of the families’ situation through the interviews. A relevant question to raise is to what extent Facebook members gave their consent to the use of their posts. The web master posted a survey about the use of posts for research purposes and based on the result of this survey consent was obtained. Using data in a closed group, removing names/nicknames and slightly modify quotes from Facebook communication to avoid identification via search engines, was considered to secure confidentiality. However, these two data sources differ in terms of validity. Firstly, because the researchers did not select individuals from the Internet-based material, this population is possibly skewed in its representation. In contrast, we were able to apply a more strategic and purposeful sampling procedure for the interviews. Secondly, it is not certain that the children referred to in the Facebook posts really have PFAPA. The patients in the interview cohort were consecutively included from patients with typical PFAPA, i.e. they fulfilled the classical criteria by Thomas et al. [2] and did not have any additional symptoms that would indicate monogenic periodic fever syndrome or unclassifiable periodic fever syndrome. Verifying emerging findings from Facebook data by further exploring these topics in the interviews helped us identify consistent patterns across the different data sources. As always in qualitative studies, transferability is dependent on contextual conditions and we claim that our inclusion of two different contexts increases transferability to a wider international population. In line with Malterud [18] we claim that in this exploratory study, we did not aim for a complete description of all aspects of experiences of parents living with a child with PFAPA in daily life. Instead, the objective was to provide insights that complement or challenge our current clinical understandings of this unexplored phenomenon.

## Conclusions

PFAPA syndrome is often considered a benign condition with spontaneous resolution. Nevertheless, this study indicates that PFAPA is associated with a considerable burden for the child and the parents in their daily life. The study emphasises the importance of obtaining a diagnosis that enables parents to move from a state of uncertainty towards a sense of coherence. The parent’s burden and efforts to care for their child and to mitigate the consequences of recurrent episodes need to be recognized and supported by healthcare professionals. Furthermore, the study highlights the importance of increased knowledge about PFAPA and what it entails for patients and their parents in the healthcare system and society at large.

## Abbreviations

PFAPA: Periodic fever, Aphthous stomatitis, Pharyngitis and cervical adenitis
